# Clinical relevance of lead-induced tricuspid regurgitation after transvenous implantable cardioverter-defibrillator implantation in arrhythmogenic cardiomyopathy

**DOI:** 10.1093/europace/euag284

**Published:** 2026-10-03

**Authors:** Federico Migliore, Raimondo Pittorru, Alberto Cipriani, Giulia Winnicki, Gaia Zancanaro, Jacopo Rosso, Sara Pisoni, Marco Previtero, Veronica Azzolin, Manuel De Lazzari, Nicolò Martini, Alessandro Rizzo, Barbara Bauce, Domenico Corrado

**Affiliations:** Department of Cardiac, Thoracic, Vascular Sciences and Public Health, University of Padova, Via Gustiniani 2, 35128 Padova, Italy; Department of Cardiac, Thoracic, Vascular Sciences and Public Health, University of Padova, Via Gustiniani 2, 35128 Padova, Italy; Department of Cardiac, Thoracic, Vascular Sciences and Public Health, University of Padova, Via Gustiniani 2, 35128 Padova, Italy; Department of Cardiac, Thoracic, Vascular Sciences and Public Health, University of Padova, Via Gustiniani 2, 35128 Padova, Italy; Department of Cardiac, Thoracic, Vascular Sciences and Public Health, University of Padova, Via Gustiniani 2, 35128 Padova, Italy; Department of Cardiac, Thoracic, Vascular Sciences and Public Health, University of Padova, Via Gustiniani 2, 35128 Padova, Italy; Department of Cardiac, Thoracic, Vascular Sciences and Public Health, University of Padova, Via Gustiniani 2, 35128 Padova, Italy; Department of Cardiac, Thoracic, Vascular Sciences and Public Health, University of Padova, Via Gustiniani 2, 35128 Padova, Italy; Department of Cardiac, Thoracic, Vascular Sciences and Public Health, University of Padova, Via Gustiniani 2, 35128 Padova, Italy; Department of Cardiac, Thoracic, Vascular Sciences and Public Health, University of Padova, Via Gustiniani 2, 35128 Padova, Italy; Department of Cardiac, Thoracic, Vascular Sciences and Public Health, University of Padova, Via Gustiniani 2, 35128 Padova, Italy; Department of Cardiac, Thoracic, Vascular Sciences and Public Health, University of Padova, Via Gustiniani 2, 35128 Padova, Italy; Department of Cardiac, Thoracic, Vascular Sciences and Public Health, University of Padova, Via Gustiniani 2, 35128 Padova, Italy; Department of Cardiac, Thoracic, Vascular Sciences and Public Health, University of Padova, Via Gustiniani 2, 35128 Padova, Italy

**Keywords:** Arrhythmogenic cardiomyopathy, Tricuspid regurgitation, Implantable cardioverter-defibrillator, Heart failure

## Abstract

**Graphical Abstract:**
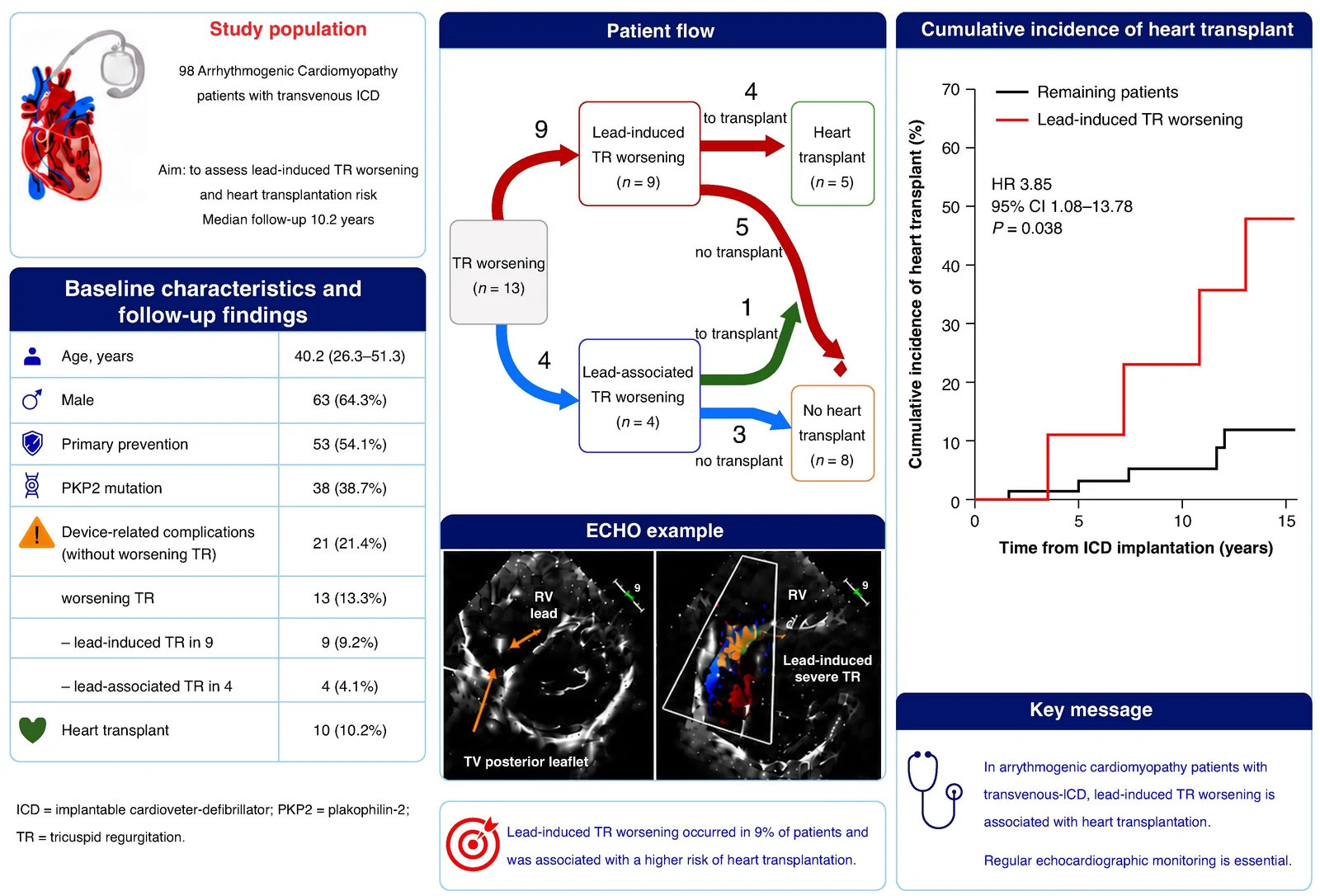

Arrhythmogenic cardiomyopathy (ACM) is an inherited myocardial disease characterized by progressive fibrofatty myocardial replacement, ventricular arrhythmias, and an increased risk of sudden cardiac death (SCD).^1^ For decades, transvenous implantable cardioverter-defibrillators (TV-ICDs) have been the standard strategy for SCD prevention in patients with ACM, including young individuals.^1^ However, transvenous leads are known to interfere with tricuspid valve function, potentially resulting in worsening tricuspid regurgitation (TR).^2–4^ Worsening of TR by at least one grade after cardiac implantable electronic device implantation has been reported in ∼10–39% of patients.^5^ Although significant TR has been associated with heart failure (HF) progression and adverse outcomes,^2-4^ the incidence and prognostic impact of lead-induced TR worsening in ACM remain poorly defined. We investigated the incidence, mechanisms, and clinical significance of TR worsening following TV-ICD implantation in patients with ACM.

We retrospectively included patients with definite ACM who underwent *de novo* TV-ICD implantation for SCD prevention between 1997 and 2024 at the University of Padua. Arrhythmogenic cardiomyopathy phenotype was retrospectively classified according to the cited criteria.^1^ The institutional review board approved the study, and all patients included provided written informed consent. Tricuspid regurgitation worsening was defined as an increase of at least one grade resulting in moderate or greater TR from the pre-implantation to the post-implantation echocardiographic assessment. Tricuspid regurgitation severity was assessed by two experienced operators using a multiparametric approach. Lead-induced TR was defined by imaging evidence of lead impingement with impaired coaptation and an eccentric jet and lead-associated TR as worsening without direct lead-leaflet interaction.^3^ The primary endpoint was heart transplantation (HT) for advanced HF despite optimal medical therapy. Time-to-event was calculated from ICD implantation to HT or last follow-up. Heart transplantation-free survival was estimated by Kaplan–Meier and compared with log-rank testing. Univariable Firth penalized Cox regression estimated HRs with 95% CIs. A two-sided *P* < 0.05 was considered significant. Analyses used SPSS version 31.

The study included 98 patients (64.3% male), with a median age of 40.2 (26.3–51.3) years at TV-ICD implantation. Arrhythmogenic cardiomyopathy phenotypes included right-dominant ACM (*n* = 32, 32.7%), biventricular ACM (*n* = 56, 57.1%), and left-dominant ACM (*n* = 10, 10.2%). Baseline characteristics are shown in *Figure 1*. During 10.2 (6.0–13.4) years of follow-up, appropriate ICD therapies occurred in 32 (32.7%) patients, inappropriate shocks in 15 (15.3%), and other device-related complications in 21 (21.4%). Tricuspid regurgitation worsening occurred in 13 (13.3%) patients: nine lead-induced and four lead-associated. Lead-induced mechanisms were septal leaflet impingement (*n* = 4), posterior leaflet impingement (*n* = 2), both leaflets (*n* = 1), or subvalvular entanglement (*n* = 2). Lead-associated TR reflected right ventricular (RV) or annular dilatation, RV dysfunction, leaflet tethering, or atrial fibrillation (AF). Tricuspid regurgitation improved after rhythm-control treatment in two patients with persistent AF. No patient underwent tricuspid valve intervention or died during follow-up. No pacing-induced TR worsening was observed. Tricuspid regurgitation worsening was similar in biventricular and right-dominant ACM [8/56 (14.3%) vs. 5/32 (15.6%); *P* = 1.0], as was lead-induced TR [6/56 (10.7%) vs. 3/32 (9.4%); *P* = 1.0]. Among patients with right-dominant or biventricular ACM, left ventricular systolic function was comparable in patients with and without lead-induced TR (*P* = 0.2). At the last available echocardiographic assessment, patients with lead-induced TR had lower tricuspid annular plane systolic excursion [11.5 (9.5–12.6) vs. 18.3 (15.1–21.8) mm; *P* < 0.001], fractional area change [20 (16–24)% vs. 29 (26–33)%; *P* = 0.001], and greater RV end-diastolic volume index [100 (90–115) vs. 82 (80–92.5) mL/m^2^; *P* = 0.001]. Time-to-event analyses were restricted to patients with right-dominant or biventricular ACM (*n* = 88). During follow-up, 10 patients underwent HT. Nine of the 10 patients who underwent HT had biventricular ACM; among the four transplanted patients with lead-induced TR, three had biventricular ACM and one had right-dominant ACM. At univariable analysis, lead-induced TR worsening was associated with an increased risk of HT (HR 3.85, 95% CI 1.08–13.78; *P* = 0.038; log-rank *P* = 0.026); see *Figure 1*.

**Figure 1:**
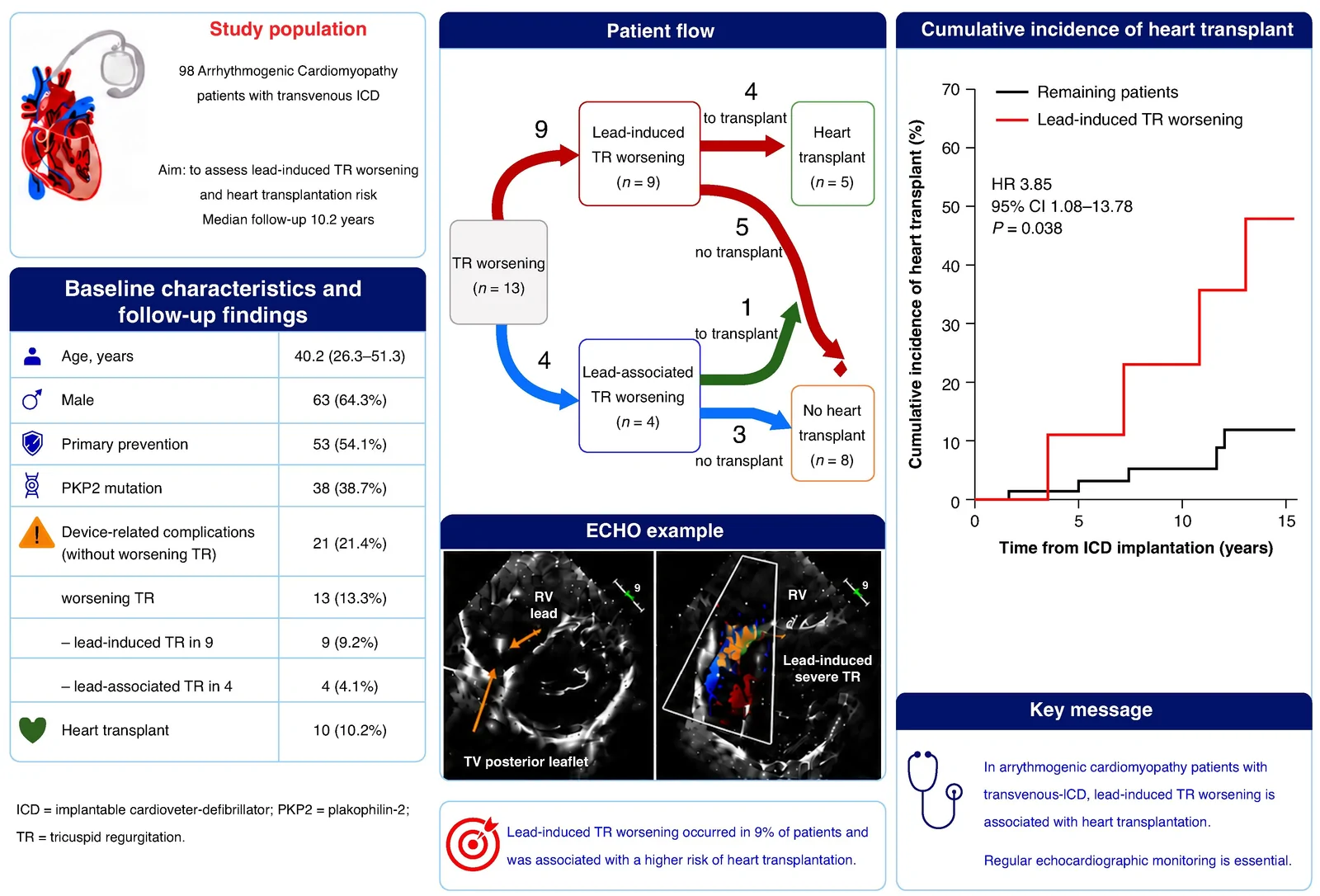
Baseline clinical, patient flow, and follow-up findings. ICD, implantable cardioverter-defibrillator; TR, tricuspid regurgitation.

The main findings are that TR worsening occurred in a relevant proportion after TV-ICD implantation and lead-induced TR was associated with HT. Although TR may reflect progression of RV disease, mechanical lead-leaflet interaction may add haemodynamic burden in susceptible patients.^6^ This extends prior evidence that significant TR in ACM carries prognostic information.^7^ Biventricular involvement represents a more advanced ACM phenotype.^8^ In our cohort, 9 of the 10 patients undergoing HT had a biventricular phenotype, supporting the severity of the underlying myocardial disease in this subgroup. Although LV systolic function and the prevalence of biventricular ACM were comparable between patients with and without lead-induced TR, patients with lead-induced TR showed greater RV dilatation and dysfunction at follow-up. This may reflect both progression of the underlying cardiomyopathy and, at least in part, the chronic haemodynamic consequences of significant TR. Lead-induced TR should therefore be considered a relevant long-term complication of transvenous ICD therapy in ACM. In the era of extravascular ICDs, this risk may inform individualized device selection.^9,10^

This study has several limitations, including its retrospective single-centre design, the lack of a comparison group treated with non-transvenous ICD systems, and the limited number of HT events. Because of the retrospective design, the exact timing and longitudinal progression of TR worsening could not be reliably assessed. The limited number of HT events precluded reliable multivariable modelling and identification of independent predictors of transplantation. Future prospective studies are needed.

In conclusion, among patients with ACM undergoing TV-ICD implantation, lead-induced TR worsening was associated with an increased risk of subsequent HT. Larger multicentre studies are warranted to confirm these findings and to determine whether the risk of lead-induced TR should influence ICD selection in patients with ACM.

## Data Availability

The data underlying this article will be shared on reasonable request to the corresponding author.
